# Outpatient Palliative Care Program: Impact on Home Death Rate in Brazil

**DOI:** 10.3390/cancers16071380

**Published:** 2024-03-31

**Authors:** Sarah Ananda Gomes, Danielle Nunes Moura Silva, Flavia Sorice, Alexandra Arantes, Rafaela Peixoto, Renata Ferrari, Matheus Martins, Alexandre Jácome, Cristiane Bergerot, Andreia Cristina de Melo, Bruno Ferrari

**Affiliations:** Oncoclinicas&Co – Medica Scientia Innovation Research (MEDSIR) /MedSir, Sao Paulo 04543-906, SP, Brazil; danielle.moura@oncoclinicas.com (D.N.M.S.); flavia.sorice@oncoclinicas.com (F.S.); alexandra.arantes@medicos.oncoclinicas.com (A.A.); rafaela.peixoto@oncoclinicas.com (R.P.); renata.ferrari@oncoclinicas.com (R.F.); matheus.martins@oncoclinicas.com (M.M.); alexandre.jacome@medicos.oncoclinicas.com (A.J.); cristiane.bergerot@oncoclinicas.com (C.B.); andreia.melo@medicos.oncoclinicas.com (A.C.d.M.);

**Keywords:** palliative care, outpatient care, terminal care, quality of life

## Abstract

**Simple Summary:**

This study investigates the impact of an outpatient palliative care program on the location of death among patients with cancer. The findings reveal a notable increase in home and hospice deaths for those participating in the program, highlighting the potential benefits of specialized palliative care in challenging healthcare contexts.

**Abstract:**

While the positive impact of early palliative care on the quality of life of cancer patients is well established, there is a noticeable research gap in developing countries. This study sought to determine the impact of an outpatient palliative care (OPC) program on the location of death among patients in Brazil. This was a retrospective study including patients with cancer who died between January 2022 and December 2022 in 32 private cancer centers in Brazil. Data were collected from medical records, encompassing demographics, cancer characteristics, and participation in the OPC program. The study involved 1980 patients, of which 32.3% were in the OPC program. OPC patients were predominantly younger (average age at death of 66.8 vs. 68.0 years old, *p* = 0.039) and composed of women (59.4% vs. 51.3%, *p* = 0.019) compared to the no-OPC patients. OPC patients had more home/hospice deaths (19.6% vs. 10.4%, *p* < 0.001), and participation in the outpatient palliative care program strongly predicted home death (OR: 2.02, 95% CI: 1.54–2.64). Our findings suggest a significant impact of the OPC program on increasing home and hospice deaths among patients with cancer in our sample. These findings emphasize the potential of specialized OPC programs to enhance end-of-life care, particularly in low-resource countries facing challenges related to social and cultural dimensions of care and healthcare access.

## 1. Introduction

Palliative care has emerged as a pivotal component in the comprehensive management of patients with cancer, demonstrating significant benefits in reducing symptoms, improving the quality of life, and even impacting overall survival. Numerous studies have underscored the positive influence of early integration of palliative care on patients, leading to a paradigm shift in the approach to cancer care [1,2,3,4]. Several studies have consistently shown that early palliative care intervention in an outpatient setting is associated with a myriad of advantages, ranging from enhanced symptom control to improvements in the emotional and psychosocial well-being of patients [5,6,7].

Recognizing these benefits, the American Society of Clinical Oncology (ASCO) has laid down guidelines emphasizing the integration of palliative care into standard oncology care [8,9]. The American Society of Clinical Oncology (ASCO) guidelines are geared towards optimizing patient-centered care by addressing the unique challenges faced by individuals navigating a cancer diagnosis. 

In alignment with the commitment to advancing the quality of palliative care, prominent organizations, such as the American Society of Clinical Oncology (ASCO), through its Quality Oncology Practice Initiative (QOPI) [10], the National Comprehensive Cancer Network (NCCN) [11], the National Consensus Project for Quality Palliative Care (NCP) [12], and the European Association for Palliative Care (EAPC) [13], have collectively established crucial quality indicators. These indicators serve as benchmarks for assessing the effectiveness and appropriateness of palliative care interventions. Key indicators encompass timely initiation of palliative care, comprehensive symptom assessment and management, effective communication and shared decision-making, documentation of advance care planning, end-of-life (EoL) care measures, including place of death, and support for patients and their families in navigating the complexities of serious illness. The integration of these indicators into clinical practice aims to ensure that palliative care is not only provided but also optimized, fostering a holistic and patient-centered approach across diverse healthcare settings. The location of death is one of these indicators, serving as a crucial measure of the quality of end-of-life care [14,15]. Additionally, death at home is associated with lower healthcare expenses and a decreased likelihood of undergoing aggressive and futile treatments [16,17].

While the merits of palliative care are well documented, the focus has predominantly been on high-income countries. Robust evidence from these settings has driven the development of guidelines and recommendations [9]. However, there is a noticeable scarcity of data from developing countries [18], particularly in regions with unique healthcare landscapes, such as Brazil. In this context, outpatient palliative care (OPC) services have gained prominence as a dynamic approach to delivering comprehensive care beyond the confines of a hospital setting. OPC extends its reach beyond mere symptom management, encompassing a spectrum of services tailored to meet the evolving needs of patients and their families. Outpatient palliative care teams have early access to patients and can monitor the progression of the disease and its implications [19]. They also have more opportunities for relationship-building [20] as well as providing education to stakeholders about the importance of avoiding futile treatments [21]. Our primary objective is to bridge the existing knowledge gap regarding the effectiveness of OPC (outpatient palliative care) initiatives, particularly within the unique context of developing nations. To this end, our study aims to assess the influence of an OPC team on the location of death among patients receiving care at private cancer centers in Brazil.

## 2. Materials and Methods

This was a retrospective cohort study performed at 32 private cancer centers located across different Brazilian states, including Central-West (*n* = 3), South (*n* = 8), Southeast (*n* = 16), and Northeast (*n* = 5). Data were collected from January to February 2023. Ethics approval was obtained from the Research Ethics Committee of Faculdade de Ciencias Medicas of Minas Gerais (protocol code: 6.121.863; date of approval: 13 June 2023). The study was conducted in clinics affiliated with the Oncoclinicas Group, Sao Paulo, SP, Brazil, a private initiative for the management and administration of oncological services founded in 2010. At the time of data collection, the group had 132 units spread across Brazil. These units cater to oncology patients with various health insurance plans and private individuals for the administration of chemotherapy, immunotherapy, radiotherapy, and clinical consultations by the oncology medical team. Additionally, they receive necessary ambulatory assistance from the multidisciplinary team.

### 2.1. Participants and Data Collection

Eligible patients were aged 18 years and older with any type of cancer who passed away between January 2022 and December 2022. Data were extracted from the patients’ medical records, where individual information was routinely documented by physicians and the multidisciplinary team at each unit. This information included date of birth, gender, type of cancer, disease stage, and the location of death. Patients were categorized into two groups based on their participation in the OPC program: OPC program participants and non-OPC program participants. The no-OPC group was followed up by cancer physicians exclusively and with multidisciplinary teams as demanded.

### 2.2. Outpatient Palliative Care Program

**Figure 1:**
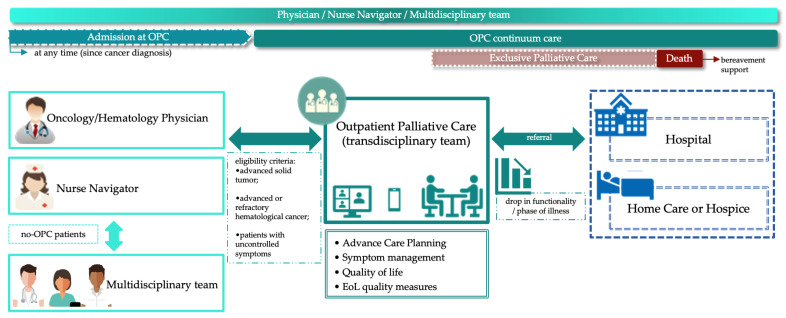
Outpatient palliative care embedded in a private cancer network in Brazil.

The OPC program follows the model proposed by Hui and colleagues [22], aimed at providing comprehensive care within an ambulatory setting (Figure 1). The program’s core objectives include preserving patient autonomy, managing symptoms effectively, and enhancing overall quality of life. This care is provided alongside oncology/hematology treatment by the patient’s attending physician. Eligibility for OPC referral encompasses patients with advanced solid tumors or clinically uncontrolled symptoms, as well as those with advanced or refractory hematological cancers. [8,23]. Referrals to OPC are typically initiated by attending physicians, with confirmation of eligibility required from the attending physician or other healthcare professionals. The OPC team primarily comprises a palliative care physician, nurse, and psychologist, complemented by additional providers such as social workers, nutritionists, pharmacists, physiotherapists, and speech-language therapists as needed. Initial appointments include the entire OPC team, and subsequent follow-ups are led by the palliative care physician alongside other team members. Family members or caregivers are actively encouraged to participate in appointments. All patients undergo monthly remote monitoring via telehealth, ensuring continuous care and support throughout their illness trajectory. Additionally, individualized care plans are developed and adjusted based on changes in patient status and clinical needs, with follow-up evaluations conducted at variable intervals capped at a maximum of 90 days.

### 2.3. Statistical Analysis

Descriptive analyses were conducted to characterize patients’ demographics (age, gender) and clinical data (type of cancer, disease stage, and location of death). For between-group comparisons, chi-squared tests were employed for categorical variables, and independent *t*-tests were used for continuous variables.

To determine the association between OPC program participation and the location of death, logistic regression analysis was performed. Only complete cases were included in the analysis. The model was adjusted for potential confounding variables, including age, gender, and type of cancer. All statistical analyses were conducted using SPSS (Version 18.0), and a significance level of *p* ≤ 0.05 was considered statistically significant.

## 3. Results

A total of 1980 patients were included in this analysis. Within the total cohort, 32.3% of patients participated in the OPC program. In the OPC group, patients had a mean age at death of 66.83 years old (SD = 16.68), while in the no-OPC group, the average was 68.06 years old (SD = 15.63) (*p* = 0.039). The patient distribution by gender revealed that 59.4% in the OPC group were female, compared to 51.3% in the no-OPC group (*p* = 0.019). Regarding cancer types, the OPC group was predominantly diagnosed with breast (22.0%), gastrointestinal (19.4%), and hematological (17.0%) cancers. Conversely, the no-OPC group exhibited higher frequencies of gastrointestinal (35.0%), thoracic (13.9%), and hematological (13.4%) cancers. The majority of patients across both groups were diagnosed with disease stage IV (76.9%, *p* = 0.241) and predominantly originated from the Southeast region of Brazil (64.2%, *p* < 0.001). Notably, the highest number of deaths attributed to cancer found in the Southeast is attributable to the greater number of cancer center units. It is noteworthy that this program was initially implemented in the Southeast (Table 1).

A higher proportion of patients enrolled in the OPC died either at home or in hospice settings compared to those in the no-OPC program (19.7% vs. 10.4%, *p* < 0.001). In logistic regression analyses adjusted for confounding variables, including age, gender, and type of cancer, the participation in the OPC program revealed an odds ratio (OR) of 2.02 (95% CI: 1.54–2.64) for home death. This underscores the predictive nature of OPC program participation in influencing the location of death.

## 4. Discussion

To our knowledge, this study marks the first Brazilian investigation into the impact of an OPC program on the location of death within private cancer centers. Conducted in 32 different centers spanning four regions in Brazil, the findings revealed a two-fold increase in the likelihood of experiencing death at home when patients are under the care of an OPC team. The choice of “place of death” as a focal point aligns with its extensive use in various studies as a quality measure in EoL care [14,15,16,17,18,19,20,21,23,24]. Information on the place of death is useful for informing the development of public policies and ensuring adequate end-of-life care for patients and bereaved survivors [25].

It is essential to recognize that the multifaceted nature of death involves factors such as socioeconomic conditions, patient preferences, access to essential resources, and medications crucial for a peaceful passing. While the current study emphasizes the importance of the place of death, we acknowledge that it is just one of several measures contributing to the assessment of end-of-life care quality. The complex interplay of these measures, including physical and psychosocial dimensions, should be considered [26]. Our decision to concentrate on the place of death is particularly relevant in the context of limited research on this aspect in Latin America, as highlighted by existing literature [27].

Despite the notable influence of the outpatient palliative care program on the location of death, it is imperative to acknowledge that the proportion of patients experiencing death at home or in hospice remains relatively low compared to the global literature, where figures hover around 50.1% [28]. This discrepancy is likely attributed to the unique cultural context of Brazil, where healthcare services predominantly follow a paternalistic model and societal attitudes towards palliative care are marked by persistent myths and misconceptions [29]. The prevailing reluctance to accept palliative care services can be a significant barrier [29], shaping EoL preferences and contributing to the observed lower rates of home and hospice deaths in this study.

In Brazil, historical factors have significantly influenced end-of-life preferences and contributed to lower rates of home and hospice deaths in our study. Despite the establishment of organizations such as the National Academy for Palliative Care, the integration of palliative care into academic curricula, particularly in medical schools, remains challenging. The Brazilian healthcare system has made gradual strides in adopting palliative care, exemplified by initiatives like the National Program for Pain Care and Palliative Care [29]. However, the private sector, especially in home care, faces ethical challenges, hindering the comprehensive incorporation of palliative care into the Brazilian healthcare system. Notably, there is a significant service gap, with only 234 palliative care services nationwide, equating to approximately 2 services per 100,000 inhabitants [30]. The private sector’s inadequacy is exacerbated by a lack of recognition and reimbursement regulations. Home care services encounter similar challenges, with only 35.4% of palliative care services offering specialized home care, and no mandatory coverage by health insurers [30]. In this context, our study introduces the OPC program as an innovative initiative, aiming to address critical gaps in palliative care delivery in Brazil. Overcoming cultural, ethical, and operational barriers is crucial for aligning palliative care services with Brazil’s unique needs, fostering greater acceptance, influencing EoL preferences, and potentially increasing the proportion of home and hospice deaths.

Building upon these observations, our findings represent a critical advancement, indicating that the OPC program holds the potential to facilitate early referral by palliative care teams to home care services. The commitment of OPC teams to establishing trust, fostering discussions about EoL care, promoting prognostic awareness, and implementing advance care planning emerges as a pivotal factor, especially in a country where such discussions remain taboo, even within the field of oncology [31]. This echoes the broader societal attitudes in Brazil, as highlighted by a cross-country study in 2017. The survey revealed distinct priorities for health care at the EoL across Japan, Italy, the United States, and Brazil, with a notable emphasis on extending life in the Brazilian context. Despite these cultural nuances, our findings underscore the transformative potential of the outpatient palliative care program, shedding light on its role in reshaping EoL preferences and potentially contributing to a higher proportion of home and hospice deaths in the future [32].

Further studies at a systematic review delving into the preferences of patients and caregivers regarding the place of death consistently revealed a strong inclination towards home as the preferred location for care and EoL [33]. This sentiment was corroborated by a recent prospective longitudinal study conducted in Brazil [24], emphasizing home as the preferred place of death. However, the study also shed light on various challenges associated with achieving death at the preferred location, particularly within the unique political and socioeconomic context of Brazil. Notably, the formal and specialized support required for dying at home is not readily available in many Latin American countries, as highlighted in a comprehensive study spanning 12 Latin American nations [34]. The scarcity of such services prompts crucial discussions for potential policy changes in the region. 

Moreover, our other recent findings for this same OPC program [35] further underscore the importance of outpatient settings. This research revealed that the average cost of hospitalization for patients who did not participate in the OPC program was significantly higher than those who received palliative care [35]. Additionally, patients admitted to the intensive care unit (ICU) incurred more costs than those who stayed in the general ward. This study highlights not only the economic benefits of providing outpatient palliative care in a developing country but also its potential to reduce healthcare costs by decreasing the length of hospital stays and preventing unnecessary ICU admissions [35]. These findings validate the shortage of human, financial, and structural palliative care resources mentioned earlier, while underscoring the importance for policymakers and healthcare providers to prioritize the provision of OPC in Brazil. It should be considered an essential component of end-of-life care.

Addressing the stigma surrounding palliative care and home/hospice deaths in Brazil is also relevant and requires a multifaceted approach. First and foremost, raising awareness about the benefits of palliative care and fostering open conversations about death are essential steps. Educating healthcare professionals, patients, and the broader community about the comprehensive support available through palliative care can contribute to dismantling misconceptions and reducing stigma. As part of our commitment to enhancing palliative care practices, we are pleased to announce a forthcoming training program in partnership with ASCO. This initiative aims to provide newly graduated oncologist physicians with specialized training in palliative care, ensuring a growing cadre of healthcare professionals equipped to deliver high-quality and compassionate end-of-life care. Additionally, initiatives promoting accessibility to essential resources, medications, and socioeconomic support can further empower individuals to make choices aligned with their preferences. While our study emphasizes the significance of the place of death as a quality measure, we acknowledge that a holistic evaluation of end-of-life care quality must consider various factors, including socioeconomic conditions, patient preferences, and resource accessibility, all crucial elements in ensuring a dignified and peaceful passage.

## 5. Limitations and Strengths

This study has inherent limitations that warrant consideration. The retrospective nature of the study introduces challenges in establishing causation, relying on historical data rather than a prospective and controlled approach. Also, due to the retrospective study design, certain variables of potential interest, such as marital status, family structure, and socioeconomic factors, were not collected, limiting the depth of analysis in these areas. Another limitation of our study is the absence of standardized classifications for types of cancer and disease stages, which makes it challenging to compare our results with those of other studies. The inclusion of a comparative group, albeit not implemented through randomized allocation, adds value to the analysis but does not provide the same level of control as a randomized controlled trial. Additionally, the study’s scope is confined to a specific context, exclusively involving patients with access to supplementary healthcare within a private oncological center in Brazil. The regional concentration of the sample, primarily from the Southeast, may introduce bias, limiting the generalizability of the results to regions with distinct historical, socioeconomic, cultural, and healthcare access dynamics. Furthermore, as our study focused exclusively on patients receiving care at private cancer centers in Brazil, the findings may not be directly generalizable to other healthcare settings or populations. This is due to differences in healthcare systems, cultural attitudes toward end-of-life care, and the availability of palliative care services. Therefore, caution is warranted when extrapolating the results to broader contexts. Moreover, it is important to acknowledge that our combined categorization of home and hospice deaths may obscure nuanced differences between these settings, potentially impacting the interpretation of end-of-life care preferences and experiences. Despite these limitations, the study shows the positive impact of specialized palliative care teams in the outpatient setting, particularly in influencing higher rates of home deaths compared to in-hospital deaths. This observation suggests a potentially effective strategy for improving the quality and humanization of the dying process, with implications for shaping public policies that comprehensively address the palliative care healthcare network, including outpatient services.

## 6. Conclusions

In conclusion, this study sheds light on the impact of an outpatient palliative care program in a private oncological center in Brazil, revealing valuable insights despite its retrospective design and absence of a randomized controlled setting for the comparative group. The findings emphasize the positive influence of specialized palliative care teams in the outpatient context, notably in increasing rates of home deaths compared to in-hospital deaths. These observations contribute to ongoing discussions about the development of inclusive public policies addressing the diverse facets of the palliative care healthcare network, particularly in outpatient settings. The study reinforces the need for further research, including prospective and multi-center studies, to validate and extend these findings, fostering a deeper understanding of the role of palliative care in shaping end-of-life experiences in diverse healthcare contexts.

## Figures and Tables

**Table 1:** Patients’ characteristics (*n* = 1980).

Variables	OPC (*n* = 640)	no-OPC (*n* = 1340)	Total Sample (*n* = 1980)	*p*-Value
Age (Mean (SD))	66.80 (16.68)	68.06 (15.63)	68.0 (15.23)	0.039
Gender (*n* (%))				0.019
Female	380 (59.4)	687 (51.3)	1067 (53.9)	
Male	260 (40.6)	653 (48.7)	913 (46.1)	
Country Regions				
Southeast	411 (64.2)	862 (64.3)	1273 (64.3)	
Northeast	90 (14.0)	253 (18.9)	343 (17.3)	<0.001
Central-West	67 (10.5)	140 (10.5)	207 (10.5)	
South	72 (11.3)	85 (6.3)	157 (7.9)	
Types of cancer (*n* (%))				<0.001
Gastrointestinal	124 (19.4)	470 (35.0)	594 (30.0)	
Breast	141 (22.0)	151 (11.3)	292 (14.8)	
Hematological	109 (17.0)	179 (13.4)	288 (14.5)	
Thoracic	72 (11.3)	186 (13.9)	258 (13.0)	
Genitourinary	50 (7.8)	178 (13.3)	228 (11.5)	
Gynecologic	63 (9.9)	61 (4.5)	124 (6.3)	
Head and Neck	31 (4.8)	40 (3.0)	71 (3.6)	
Central Nervous System	12 (1.9)	23 (1.7)	35 (1.8)	
Others	38 (5.9)	52 (3.9)	90 (4.5)	
Disease Stage (*n* (%))				0.241
I	7 (1.1)	14 (1.0)	21 (1.0)	
II	18 (2.8)	33 (2.5)	51 (2.6)	
III	43 (6.7)	79 (5.9)	122 (6.2)	
IV	453 (70.8)	1005 (75.0)	1458 (73.7)	
Hematological	83 (13.0)	127 (9.5)	210 (10.6)	
Unknown	36 (5.6)	82 (6.1)	118 (5.9)	
Place of Death				
Home/Hospice	125 (19.5)	139 (10.4)	264 (13.3)	<0.001
Hospital	511 (79.8)	1198 (89.4)	1709 (86.3)	
Unknown	4 (0.7)	3 (0.2)	7 (0.4)	

Note: OPC, outpatient palliative care.

## Data Availability

The data presented in this study are available on request from the corresponding author. The data are not publicly available due to privacy and ethical restrictions of Oncoclinicas Group.
